# Point of Care Ultrasound of the Hemodialysis Vascular Access

**DOI:** 10.24908/pocus.v7iKidney.15348

**Published:** 2022-02-01

**Authors:** Larissa Kruger Gomes, Het Patel, Nikhil Agrawal, Yael Vin

**Affiliations:** 1 Renal Division, Department of Internal Medicine, Beth Israel Deaconess Medical Center, Harvard Medical School Boston, Massachusetts USA; 2 Division of Transplant Surgery, Beth Israel Deaconess Medical Center, Harvard Medical School Boston, Massachusetts USA

**Keywords:** point of care ultrasound, POCUS, hemodialysis vascular access, fistula, graft

## Abstract

The effectiveness of hemodialysis is completely dependent on the quality of the patient’s vascular access; thus, appropriate assessment of ateriovenous fistulas and grafts is of critical importance. Point of care Ultrasound (POCUS) can be an additional useful skill at the bedside for assessment of the hemodialysis vascular access. In this paper we discuss the basic terminology and techniques that can be employed in the POCUS exam of vascular accesses. We also delineate the current recognized criteria for access maturation and common issues that can be diagnosed with the support of POCUS. 

## Introduction

Hemodialysis is the most prevalent mode of renal replacement therapy in the United States 1. For hemodialysis to be an effective treatment relies heavily on the quality of the patient’s vascular access. To promote the success of a patient’s vascular access it is required that the clinician is well versed in maturation assessment, strategies for cannulation, monitoring, and diagnosis of potential access related issues and complications. While clinical signs and physical exam strategies are extremely important, point-of-care ultrasound (POCUS) is an additional valuable skill in order to provide optimal vascular access care. A focused ultrasound of the vascular access with a portable inexpensive device can provide important information in relatively little time.

In this review we aim to discuss the clinical applications and describe the different techniques involved in POCUS assessment of vascular access in hemodialysis patients. 

**Figure 1:**
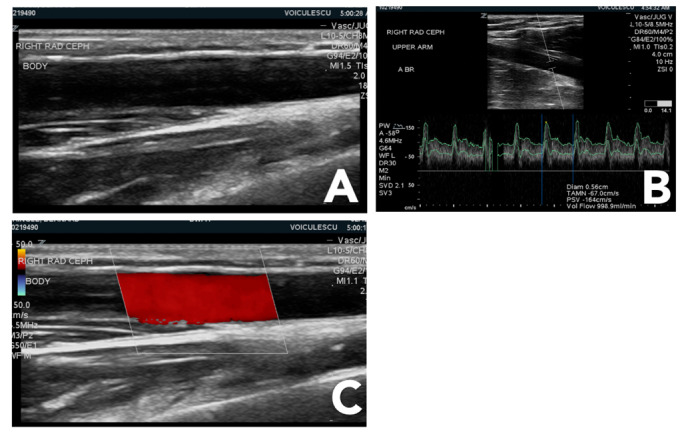
Ultrasound modes used for vascular access examination. A) B-mode (brightness); B) D-mode (Doppler); C) C-mode (color); Figures courtesy of Dr. Adina Voiculescu.

For POCUS of the vascular access, a linear phased array probe is preferred, as the focus is mostly in superficial structures. The examiner will use B mode, color Doppler, and pulsed-wave Doppler modes (Figure 1). 

B-mode is used to identify the access and other surrounding structures such as blood vessels or fluid collections. Color Doppler is used to assist and confirm findings on the B-mode and to monitor the patency of the access. Examples include showing the presence of a non-occlusive thrombus, detection of aneurysms and pseudoaneurysms, and identifying active bleeding. Doppler mode is used to measure blood flow. 

**Figure 2:**
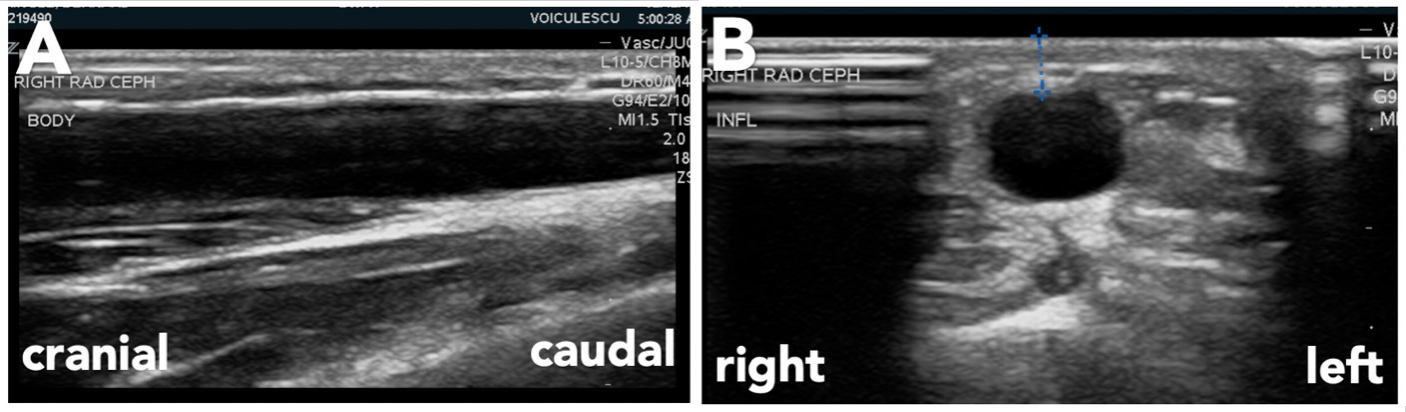
Ultrasound planes: A) longitudinal axis; B) transverse axis. Figures courtesy of Dr. AdinaVoiculescu.

When evaluating any access either by physical exam and POCUS, the operator should focus on 3 elements: inflow, conduit, and outflow. Inflow segment includes inflow artery, arterial anastomosis, and juxta-anastomotic segment. The body of the fistula or graft itself is the conduit, also termed the “stick zone”—the segment that is being cannulated. The outflow segment starts at the venous anastomosis in a graft and at the outflow veins beyond the cannulation zone in an AV fistula. In the text, when we say “longitudinal axis” we will be referring to the view in which the probe is parallel to vessel/access. When we say “transverse axis” we will be referring to the view in which the probe is perpendicular to the vessel/access. See Figure 2 for reference below. 

## Access Mapping

Pre-operative ultrasound mapping is recommended before every access creation. We will not review the vessel mapping done to guide access choice and surgical planning. We will focus on the post creation mapping of the access. The intent of this type of mapping is to determine maturation, help the dialysis unit with early cannulation attempts, and assist when difficulty with cannulation is encountered.

By using POCUS in conjunction with one’s physical exam it is possible to mark the course or path of the access with a skin marker. Markings should include the size and depth at different points in addition to identify branch veins. Such markings will make the cannulation process easier and often increase the usable area of the access 2. Additional papers have also reported a shorter time from surgery to first successful access cannulation via the use of POCUS for mapping and, thus, decreasing the need for initial placement and duration of use of tunneled dialysis catheters 2. Finally, the use of POCUS prior to cannulation has also been shown to improve the patient’s experience, decreasing the need for multiple attempts at cannulation and infiltration events 3.

In order to map a fistula, the patient should ideally be positioned at 45º and the operator should have access to the whole arm 4. The operator should scan the arm starting by focusing on the arterial inflow area in a transverse position with the ultrasound in B-mode. The two most commonly used arteries for vascular access are the radial artery (distal or proximal) and the brachial artery. The operator should follow the trajectory of the arterial vessel until the antecubital fossae if the access is distal radial (with anastomosis close to the wrist). If the access is brachiocephalic, brachiobasilic, brachio-brachial, or proximal radial (just below the elbow), the vessel should be followed until the axillary area 5. On the ultrasound monitor, the arterial vessel should be oval/round and have an even, smooth surface. 

Now that the arterial inflow has been examined in the transverse view, the evaluation of the longitudinal view should be completed—the access blood flow will be measured in this step. Although the access flow can be measured directly in the fistula or the graft, it is more accurate when measured in the inflow artery. The brachial artery should be used to measure flow in brachial artery fistulas as well as radial artery fistulas. This is because radial artery fistulas commonly get additional flow into from the ulnar artery via the palmar arch 3. 

Pulsed-wave Doppler mode is used for flow measurement. To accurately measure the flow, the operator will need to accurately perform 3 steps: obtain an appropriate angle, proper vessel diameter measurement, and selection of cardiac cycles. It is important to establish the correct angle between the Doppler beam and blood flow direction (angle of insonation). The angle of insonation should be 60º or below, otherwise the measurement will be inaccurate with steeper angles overestimating flow. For a detailed review on the physics behind this see 5. The angle of insonation is usually annotated on the right corner of the US screen. 

To obtain an appropriate angle of insonation, the operator should adapt the position of the probe, select a portion of the vessel that has an advantageous angulation, or position and use the steer button to select a good doppler window. The next step should be to measure the diameter of the access. The final step is to select the span of cardiac cycles that the ultrasound will use to calculate the flow. One should obtain measurements over 2-5 cardiac cycles and report its average 6. 

Now the venous outflow area should be examined. Just as with the arterial examination, positioning the transducer for a transverse view should be the first step. The operator should scan the vessel area looking for branching vessels or other changes. While the operator moves the probe, they should demarcate the access on the skin with a skin marker in order to “map” the access. After the first scanning through the region with a transverse view is completed, a longitudinal view should also be done in order to help with the mapping or confirm your findings.

**Figure 3:**
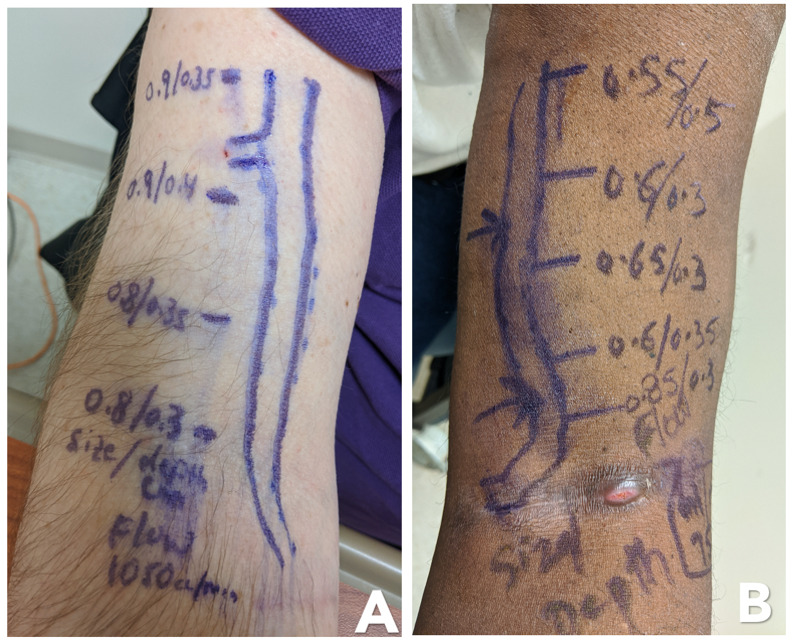
Two examples of access mapping. The operator has drawn the direction and overall shape of the access and also measured its diameter (size) and depth at different location as well as average flow. In image A you can see the operator has identified a branching vessel. In image B the operator has signaled with arrows what they believe to be the optimal points for needle insertion.

In conjunction with physical exam, the access is now drawn on the skin. Size and depth of the access should be measured at several points along the course of the access. These measurements are made in B-mode in transverse axis. It is crucial to hold the probe as gently as possible and allow access to be visualized as a full circle. Pressure on the access from the probe can markedly affect the size measurement. Abnormal findings such as hematomas or pseudoaneurysms should be marked as well. See examples of mapped fistulas in Figure 3. 

The best area for cannulation can be determined by the size and depth of the access. Ideally one would select a large superficial and straight segment with no overlying veins and optimally no nearby branching vessels. Generally, the access should be at least 5mm in diameter and less than 6mm deep in order to be optimally cannulated 7.

## Maturation Assessment

In order to ensure that a vascular access is ready and is likely to deliver appropriate access to hemodialysis treatments, the physical exam should be coupled with POCUS assessment. 20%–60% of AVFs fail to mature for successful dialysis use 6. POCUS does not replace the physical exam but enhances it. For example, while an access may meet the ultrasound criteria for maturation, it may not be clinically ready for cannulation and vice versa. 

Maturation assessment is very similar to the mapping described above but takes into consideration the nuances of a new access. Post-surgical fluid collections such as seromas and hematomas are common and should be identified. Another consideration is that a new fistula often has branch veins which can affect maturation and these should be identified for safe cannulation and potential branch vein ligation. 

There are three measurements that determine maturation by ultrasound criteria: blood flow, access depth, and access size. Different criteria or cut-offs for these measurements have been proposed and investigated.

Currently there are two adopted sets of ultrasound maturation criteria. The “rule of 6s” was first described in KDIGO guidelines in 2006 as an expert opinion and is still commonly used. It involves the following parameters: blood flow > 600 mL/min, access depth < 6 mm from the surface, and external diameter > 6 mm 8. The second set of parameters is the UAB criteria: blood flow > 500 mL/min and vein diameter > 0.4 cm. In a retrospective analysis of 69 patients who were 2-4 months post fistula creation, when both thresholds were exceeded, 95% (19 of 20) of the fistulas were adequate for dialysis. When neither threshold was achieved, only 33% (five of 15) of fistulas were adequate. Achieving just one of the two threshold values was associated with intermediate adequacy rates of 67%-70% 9. Both KDOQI and UAB criteria were validated in the hemodialysis fistula maturation study (HFM) 10. The HFM study was a large multicenter observational study, with over 600 patients which showed that AVF blood flow, diameter, and depth at 6 weeks post-surgery predicted AVF clinical maturation. The other factors that were considered included upper arm arterial diameter, presence of stenosis, presence of accessory veins, case-mix factors (age, sex, black race, AVF location, diabetes, dialysis status, and body mass index), and clinical center and did not further improve the AVF maturation prediction 10. As one might expect, the UAB criteria was more sensitive but less specific than the rule of 6s in predicting maturation.

The technique and steps to mark the fistula, identify branch veins and fluid collections, measure blood flow, and measure fistula depth and size are described above in mapping. 

## High Flow Fistula

Blood flow measurement be easily established via POCUS when a high flow access is suspected. Here POCUS is superior to physical exam alone because, while history might spark the diagnostic possibility, there are no specific physical exam findings and the direct measurement of access flow is required for a final diagnosis. 

In patients with heart failure, pulmonary hypertension, vascular steal, or central venous stenosis, measurement of access flow is critical. Presence of high flow could be a major contributing factor and could be relatively easy to correct via banding or revision surgery. In absence of these conditions, it may be prudent to reduce flow in the fistula as a prophylactic measure to prevent long term adverse cardiac effects such as high output heart failure. 

What is a high flow access is not well defined and is often relative to clinical picture. In patients with above listed conditions, any flow in excess to what is required for dialysis can be argued as high flow. In asymptomatic individuals, blood flow (Qa) of ≥ 2.0 L/min or Qa/Cardiac output ratio of >20% should prompt a discussion about prophylactic reduction in blood flow 11.

## Vascular Steal

The diagnosis of vascular steal is predominantly clinical. The physical exam maneuver of compressing the access and then evaluating symptoms continues to be the recommended initial step to identify potential vascular steal. However, physical exam does not show the anatomical culprit of the issue. POCUS can be helpful in two ways: there might be signs of flow reversal when in doppler mode and, most importantly, vascular flow might be decreased.

## Access Stenosis

Access stenosis can be assessed by POCUS via greyscale imaging in addition to pulsed-wave Doppler. The initial step is to use B-mode and search for presence of luminal narrowing. A narrowing of >50% of the access, when compared to the normal vasculature located upstream from the affected portion, is considered a positive finding. This can be done using the longitudinal or sagittal view. The second step is to determine if a peak systolic velocity (PSV) ratio between the anastomosis area of the access and the feeding artery 2 cm upstream from the anastomosis. This is done on longitudinal view. A PSV ratio >3:1 has been shown to be a very sensitive measure to suggest the presence of a stenosis. As discussed above, the measurement of flow and velocity in an access can be difficult, so if a PSV ratio is present but no stenosis is seen a fistulogram should be considered 4. 

## Hematomas, Seromas, and Abscesses

Infiltration during cannulation or due to movement of the needle during dialysis is common and can cause a hematoma. This can cause difficulty in cannulation of the access due to overlaying edema and fluid collection. In such a situation, use of POCUS in B-mode can localize areas of soft tissue swelling and fluid collection and identify sites that are safe for cannulation. The hematoma can also be measured and/or marked to allow for monitoring with future POCUS evaluation. Color Doppler can help identify active bleeding which should be promptly addressed. 

In patients suspected to have access infection, B-mode POCUS should be performed to look for fluid collection. A fluid collection in this setting may be an abscess that needs to be drained.

In post-operative patients, fluid collection is commonly due to seroma. These are less inflammatory and appear less complex on ultrasound. POCUS in B-mode can identify and localize these collections and help with cannulation and monitoring.

## Summary

Point-of-care ultrasound is an invaluable skill in the exam of dialysis vascular access. This inexpensive imaging modality can help guide proper cannulation and, in conjunction with the physical exam, establish access readiness and diagnosis of common issues. POCUS has been shown not only to improve patient’s experience, but also decreases reliance on tunneled catheters and aids in accurate and timely diagnosis of many access related complications. Nephrologists and training programs should familiarize themselves with this technological tool so that we can continue to grow as a patient focused specialty. 

## Disclosures

None.
